# Warum das C-reaktive Protein beim SLE (meist) nicht hoch ist

**DOI:** 10.1007/s00393-026-01779-4

**Published:** 2026-01-27

**Authors:** Erik Klapproth, Martyna Hempel, Nicolai Leuchten, Stefan Rose-John, Adelheid Korb-Pap, Martin Aringer

**Affiliations:** 1 Institut für Pharmakologie, Medizinische Fakultät Carl Gustav Carus, Technische Universität Dresden, Dresden, Deutschland; 2 Bereich Rheumatologie, Medizinische Klinik und Poliklinik 3, Universitätsklinikum und Medizinische Fakultät Carl Gustav Carus, Technische Universität Dresden, Fetscherstraße 74, 01307 Dresden, Deutschland; 3 UniversitätsCentrum für Autoimmun- und Rheumatische Erkrankungen (UCARE), Universitätsklinikum und Medizinische Fakultät Carl Gustav Carus, Technische Universität Dresden, Dresden, Deutschland; 4 Institut für Biochemie, Medizinische Fakultät, Christian-Albrechts-Universität zu Kiel, Kiel, Deutschland; 5 Institut für Muskuloskelettale Medizin, Universitätsklinikum Münster, Münster, Deutschland

**Keywords:** Systemischer Lupus erythematodes, Shedding, Interleukin‑6, Typ-I-Interferone, Zytokinrezeptoren, Systemic lupus erythematosus, Shedding, Interleukin‑6, Interferons, type 1, Cytokine receptors

## Abstract

Hohe Werte von C‑reaktivem Protein (CRP) sind beim systemischen Lupus erythematodes in der Regel nicht Zeichen der Krankheitsaktivität, sondern einer schweren bakteriellen Infektion. Warum das so ist, obwohl die Spiegel von Interleukin(IL)-6, dem wichtigsten CRP-Stimulator, beim aktiven SLE hoch sind, ist seit Längerem eine viel diskutierte Frage. Neu publizierte Ergebnisse geben jetzt eine Antwort darauf: In Kombination mit Interferon alpha (IFN-α) führt IL‑6 dazu, dass der IL-6-Rezeptor „geshedded“, also enzymatisch von der Zellmembran abgeschnitten wird. Die löslichen Rezeptoren im Plasma bilden einen IL-6-Puffer, der IL‑6 an Effekten auf Leberzellen hindert. Daher wird dort kein CRP produziert. Bei extrem hohen IL-6-Spiegeln im Rahmen von Infektionen (und selten Lupusserositis oder -arthritis) wird die Pufferkapazität überschritten, und die Hepatozyten reagieren durch Produktion von CRP.

## Abstract (en)

High levels of C‑reactive protein (CRP) in systemic lupus erythematosus are not usually a sign of disease activity but more of a severe bacterial infection. Why this is the case, even though levels of interleukin‑6 (IL-6) the most important CRP stimulator, are high in active SLE, has long been an intriguing question. Newly published results now provide an answer: in combination with interferon-alpha (IFNα), IL‑6 causes the IL‑6 receptor to be shed, i.e. enzymatically cleaved from the cell membrane. The soluble receptors constitute an IL‑6 buffer that inhibit IL‑6 biological activity in plasma. As this prevents IL‑6 from stimulating liver cells, no CRP is induced there. When IL‑6 levels are extremely high in the context of infections (and rarely lupus serositis or arthritis), the buffering capacity is exceeded and the hepatocytes respond by producing CRP.

C‑reaktives Protein (CRP) ist bei vielen entzündlich-rheumatischen Erkrankungen ein wertvoller Aktivitätsparameter. Das gilt jedoch nicht für den systemischen Lupus erythematodes (SLE) [1, 2]. Zwar kann CRP im Rahmen einer Lupusarthritis oder einer Lupusserositis deutlich erhöht sein [3, 4]. In der Regel deutet ein relevant erhöhtes CRP bei Patientinnen und Patienten mit SLE jedoch auf eine bakterielle Infektion und nicht auf SLE-Aktivität hin, insbesondere bei CRP-Werten, die größer als 70 mg/l (7 mg/dl) sind [1, 5].

Die CRP-Produktion in den Hepatozyten wird direkt durch IL‑6 stimuliert [6, 7]. Deshalb wäre ein niedriger IL-6-Spiegel trotz SLE-Aktivität eine hypothetisch mögliche Erklärung gewesen. Dies ist allerdings nicht zutreffend: Es besteht eine positive Korrelation zwischen der SLE-Aktivität und IL-6-Spiegeln [8, 9]. Zudem haben gegen IL‑6 gerichtete therapeutische Ansätze wie Tocilizumab oder der Jak-Inhibitor Upadacitinib in SLE-Studien eine klinische Wirksamkeit gezeigt [10, 11].

Auch Autoantikörper gegen CRP, die bei manchen SLE-Patientinnen und Patienten gefunden wurden, wurden als Ursache des fehlenden CRP-Anstiegs diskutiert [12]. Diese Antikörper sind aber einerseits relativ selten nachweisbar, und andererseits würden sie dann auch den CRP-Anstieg im Falle einer Infektion verhindern, sodass auch diese Erklärung nicht ausreichend ist. Eine skandinavische Gruppe hatte bereits den Einfluss von Typ-I-Interferonen auf eine verminderte CRP-Produktion gezeigt [13, 14]. Typ-I-Interferone wie INF‑α spielen beim SLE eine relevante Rolle; Anifrolumab, das den Typ-I-Interferon-Rezeptor blockiert, ist rasch wirksam und für die Therapie des SLE zugelassen [15].

## Der IL-6-Rezeptor

Im Rahmen einer durch die DFG geförderten Untersuchung der IL-6-Signaltransduktion konnten wir nun die Mechanismen aufdecken, wie Typ-I-Interferone das CRP beim SLE unterdrücken [16]. Für die Erklärung ist es notwendig, das Grundwissen um den IL-6-Rezeptorkomplex aufzufrischen. Der IL-6-Rezeptor besteht aus einer IL-6-Rezeptor-α-Kette (CD126) und zwei Ketten des Glykoproteins gp130 (CD130), das auch als Bestandteil anderer Rezeptoren eine Rolle spielt und auf allen kernhaltigen Zellen vorkommt [17]. CD126 findet sich hingegen nur auf drei Zellsorten, nämlich auf Leukozyten, Hepatozyten und Darmwandepithelzellen, wobei Letztere für die aktuelle Frage keine Rolle spielen.

**Abb. 1:**
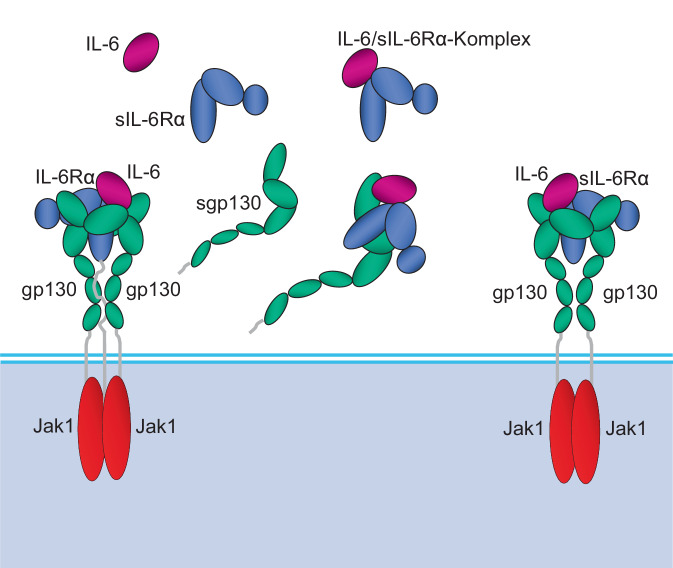
Unterschiedliche Zustände des IL-6/IL-6-Rezeptor-Komplexes. Der membranständige IL-6-Rezeptor (*IL-6R*) auf Leukozyten, Hepatozyten und Darmwandepithelzellen besteht aus einer Kette IL-6Rα und zwei Ketten gp130, die dimerisieren und jeweils ein Molekül Januskinase‑1 (*Jak1*) binden. Jak1 ist für die Signaltransduktion verantwortlich (*links*). IL-6Rα kann (durch die Metalloproteinasen ADAM10 und ADAM17) extrazellulär abgeschnitten werden. Durch dieses *Shedding* entsteht so löslicher sIL-6Rα (*Mitte oben*). Aus sIL-6Rα und IL‑6 kann sich ein Komplex binden, der über gp130-Moleküle auf jedweden kernhaltigen Zellen einen voll funktionsfähigen IL-6R für das sog. *Trans-Signaling* von IL‑6 bilden kann (*rechts*). Ist zusätzlich, ebenfalls durch Shedding, lösliches sgp130 in größeren Mengen vorhanden, bilden sich auch IL-6/sIL-6Rα/sgp130-Komplexe, die als IL-6-Puffer wirken (*Mitte unten*)

Sowohl CD126 als auch CD130 können durch Proteinasen von der Zellwand abgeschnitten werden [18]. Dieser Prozess heißt auf Englisch *Shedding*. Leukozyten oder Hepatozyten verlieren dadurch ihre Oberflächenrezeptoren, während die Serumkonzentration an löslichem (solublem) IL-6-Rezeptor‑α (sIL-6Rα) bzw. löslichem gp130 (sgp130) ansteigt. IL‑6 kann sIL-6Rα binden, und dieser Komplex (*Mitte oben* in der Abb. 1) kann wieder an CD130-Moleküle auf kernhaltigen Zellen (z. B. Endothelzellen) binden und so zum sog. *Trans-Signaling* führen [18].

Auch Zellen, die selbst kein membranständiges CD126 an der Oberfläche tragen, können so mit Hilfe von sIL-6Rα (*rechts* in Abb. 1) praktisch auf die gleiche Art IL-6-Signale übermitteln wie Leukozyten oder Hepatozyten über ihre membranständigen Rezeptoren (*links* in Abb. 1). Andererseits kann der IL-6/sIL-6Rα-Komplex durch lösliches sgp130 abgepuffert werden, wenn sowohl sIL-6Rα als auch sgp130 in größeren Mengen vorliegen (*Mitte unten* in Abb. 1). Dadurch werden IL-6-Signale und ihre biologischen Effekte verhindert.

## Die Situation bei aktivem SLE

Zunächst konnten wir in der Arbeit bestätigen, dass der IL‑6 Spiegel-positiv mit der SLE-Aktivität im European Consensus Lupus Activity Measure (ECLAM) korreliert, CRP jedoch nicht [16]. Bei der Analyse von IL-6Rα bei SLE stellte sich heraus, dass die membrangebundene Form (CD126) aktivitätskorreliert vermindert und Serum-sIL-6Rα erhöht war. Das führte auch dazu, dass die Lymphozyten der SLE-Patientinnen und -Patienten auf kurzzeitige Stimulation mit IL‑6 weniger Stat3 phosphorylierten – die JAK1-Aktivität war also vermindert [16]. Dieser Shift von membranständigem CD126 zu sIL-6Rα legte ein *Shedding* nahe.

In Stimulationsexperimenten gelang es in der Folge, die SLE-Situation in vitro nachzustellen. Aus dem peripheren Blut gewonnene mononukleäre Zellen (PBMC) in Kultur über 24 h wurden mit der Kombination aus IFN‑α und IL‑6 stimuliert, während diese Zytokine einzeln nur einen geringen und die meisten anderen getesteten Zytokine keinen Effekt hatten. IFN‑α und IL‑6 führten in Kombination zu einer deutlichen Abnahme von CD126 auf den Lymphozyten [16].

In der Folge konnten wir auch zeigen, dass diese Abnahme tatsächlich auf dem *Shedding* von IL-6Rα beruht: Auch wenn ELISA-Tests in Anwesenheit von IL‑6 nicht funktionierten, konnten wir mittels Immunpräzipitation zeigen, dass sIL-6Rα im Überstand der mit IFN‑α und IL‑6 stimulierten PBMC anstieg. Der gleiche Mechanismus war auch in einer humanen Hepatom-Zelllinie nachvollziehbar [16]. Wurden Zellen mit einem *Shedding*-resistenten IL-6Rα-Konstrukt transfiziert, folgte auf IFN-α- und IL-6-Stimulation auch kein Anstieg des sIL-6Rα im Überstand.

Bei SLE-Aktivität führt die Kombination aus IFN‑α (oder anderen, über den gleichen Rezeptor wirkenden Typ-I-Interferonen) und IL‑6 also dazu, dass Leukozyten und Hepatozyten über die Aktivierung der Metalloproteinasen ADAM10 und ADAM17 ihr membranständiges CD126 *shedden*, was zu einer Verminderung CD126-positiver Zellen bei gleichzeitiger Zunahme von sIL-6Rα führt. Statt über den konventionellen IL-6R-Leukozyten zu aktivieren und Hepatozyten zur Produktion von CRP anzuregen, bindet IL‑6 in dieser Situation vor allem an sIL-6R. Dieser IL-6/sIL-6Rα-Komplex ist in der Lage, an membranständige gp130-Moleküle z. B. von Endothelzellen zu binden und führt dort zu *Trans-Signaling*. Zudem ist aber auch sgp130 beim SLE erhöht [19], sodass der Komplex mit sIL-6Rα und sgp130 zu einer Pufferung von IL‑6 führt [20].

## Erklärung der CRP-Dynamik beim SLE

**Abb. 2:**
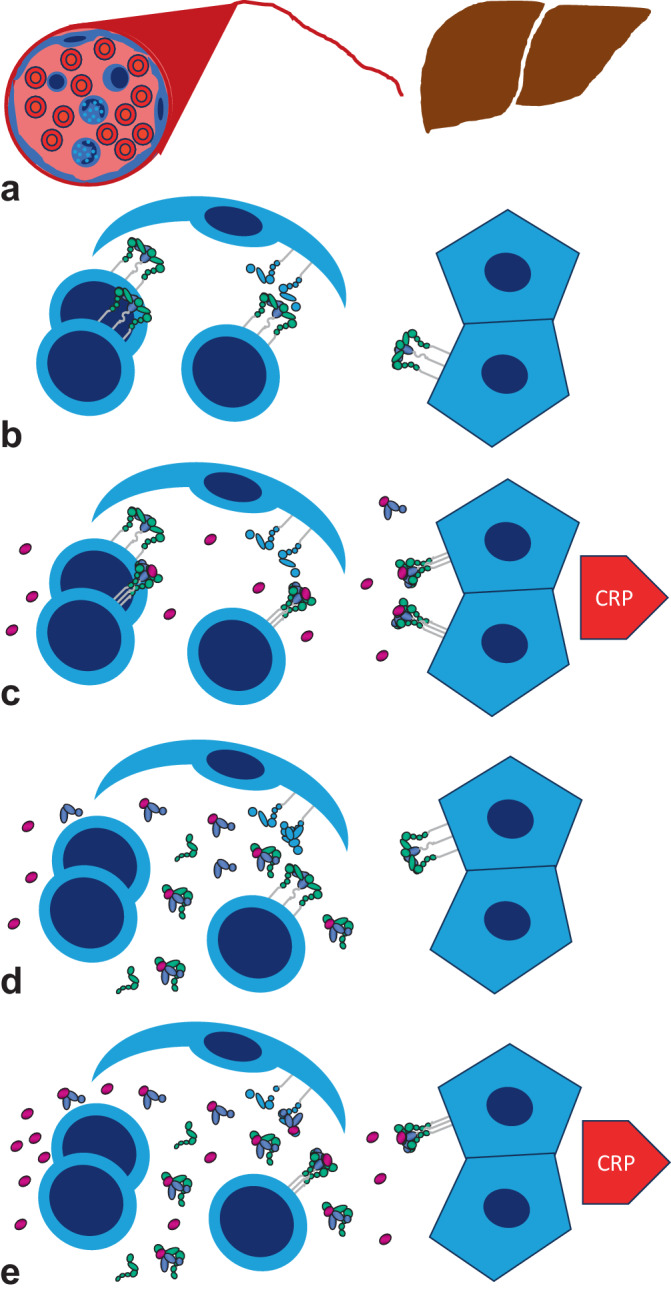
IL-6-Signaltransduktion im Grundzustand und beim SLE. Im Grundzustand (**a**) sind vor allem membranständige IL-6-Rezeptoren auf Leukozyten (*links*) und Hepatozyten (*rechts*) vorhanden, die jeweils aus zwei Molekülen gp130 (*grün*) und einem Molekül IL-6Rα (*blau*) bestehen. Auch bei Produktion relativ geringer Mengen IL‑6 (*rot-violett*) kommt es zum CRP-Anstieg (**b**). Beim aktiven SLE führen Typ-I-Interferone und IL‑6 zum *Shedding* von IL-6Rα zu sIL-6Rα, das in Kombination mit sgp130 geringe bis mäßige Mengen IL‑6 abpuffert (**c**). Sehr hohe IL-6-Mengen übersteigen die Pufferkapazität (**d**). IL‑6 trifft wieder auf Hepatozyten und führt zur CRP-Produktion

In Abb. 2 wird versucht, die Konsequenzen dieser Veränderungen beim SLE auf die CRP-Produktion darzustellen. Im Normalzustand befinden sich membranständige CD126-Moleküle auf Leukozyten und Hepatozyten (Abb. 2a). Wird IL‑6 produziert und freigesetzt, kann IL‑6 die Leber rasch erreichen und den membranständigen Rezeptor auf Hepatozyten binden. Dies führt direkt zur Produktion von CRP (Abb. 2b).

Beim SLE führt die vermehrte Bildung von Typ-I-Interferonen (IFN‑α und vermutlich IFN-κ) und IL‑6 zum *Shedding* von CD126 zu sIL-6Rα – also einer Reduktion der membranständigen bei deutlich erhöhten löslichen Rezeptoren. Der IL-6/sIL-6Rα-Komplex kann zum *Trans-Signaling* auf gp130-tragenden anderen Zellen führen. Bei gleichzeitig erhöhtem sgp130 wird IL‑6 in einem sIL-6Rα/sg130-Komplex abgepuffert. Die zudem verminderten IL-6-Rezeptoren in der Leber werden vom vorhandenen IL‑6 praktisch nicht mehr erreicht, es wird kaum CRP produziert (Abb. 2c).

Wird hingegen bei schweren bakteriellen Infektionen oder auch im Rahmen einer Lupusserositis oder -arthritis exzessiv IL‑6 produziert, übersteigt das die Pufferkapazität des sIL-6Rα/sg130-Komplexes. Es ist also deutlich mehr IL‑6 als sIL-6Rα und zumindest als sgp130 vorhanden, und freies IL‑6 wird wieder bis in die Leber transportiert, um dort über die restlichen membranständigen Rezeptoren oder auch als *Trans-Signaling* über IL-6/sIL-6Rα-Komplexe Hepatozyten zu stimulieren; CRP steigt dadurch stark an (Abb. 2d).

## Fazit

Diese neuen Ergebnisse liefern eine mechanistische Lösung für ein lange ungelöstes Rätsel: Warum steigt das CRP beim aktiven SLE trotz deutlich erhöhter Spiegel von IL‑6, dem zentralen Treiber der CRP-Produktion, meist nicht entsprechend an? In Kombination mit IL‑6 führen die beim SLE regelhaft erhöhten Typ-I-Interferone zum *Shedding* von IL-6-Rezeptoren, die zu *Trans-Signaling* und einer IL-6-Abpufferung führen. Nur wenn diese Pufferkapazität durch sehr hohe IL-6-Mengen, beispielsweise bei bakteriellen Infektionen, überschritten wird, erhalten Hepatozyten IL-6-Signale und produzieren CRP. Im Resultat wird unter Typ-I-Interferon-Einfluss die CRP-Synthese eingeschränkt. Der entwicklungsgeschichtliche Hintergrund hierfür könnte sein, dass CRP in der Abwehr von Virusinfektionen, im Gegensatz zu bakteriellen Infektionen, keine Funktion hat und damit eine Ressourcenverschwendung darstellen könnte. Dieser Mechanismus dürfte auch bei anderen Kollagenosen und eventuell in bestimmten Situationen bei der rheumatoiden Arthritis eine Rolle spielen.

## Interessenkonflikt

M. Aringer gibt Advisory boards und/oder Vorträge für AbbVie, AstraZeneca, Chugai, Galapagos, GSK, Lilly, Pfizer und Roche und Sanofi an. N. Leuchten: Advisory boards und Vorträge für Roche. Stefan Rose-John gibt Beratungstätigkeit und Vorträge für AbbVie, Chugai, Genentech Roche, Regeneron, Pfizer und Sanofi an, ist Erfinder auf Patenten im Eigentum des CONARIS Research Institutes (Ko-Entwickler des sgp130Fc-Proteins Olamkicept gemeinsam mit Ferring Pharmaceuticals und I-Mab Biopharma), und hält Aktien von CONARIS. E. Klapproth, M. Hempel und A. Korb-Pap geben an, dass kein Interessenkonflikt besteht.
